# Susceptibility and Gene Interaction Study of the Angiotensin II Type 1 Receptor (AGTR1) Gene Polymorphisms with Non-Alcoholic Fatty Liver Disease in a Multi-Ethnic Population

**DOI:** 10.1371/journal.pone.0058538

**Published:** 2013-03-06

**Authors:** Shamsul Mohd Zain, Zahurin Mohamed, Sanjiv Mahadeva, Sanjay Rampal, Roma Choudhury Basu, Phaik-Leng Cheah, Agus Salim, Rosmawati Mohamed

**Affiliations:** 1 The Pharmacogenomics Laboratory, Department of Pharmacology, Faculty of Medicine, University of Malaya, Kuala Lumpur, Malaysia; 2 Department of Medicine, Faculty of Medicine, University of Malaya, Kuala Lumpur, Malaysia; 3 Julius Centre University of Malaya, Department of Social and Preventive Medicine, Faculty of Medicine, University of Malaya, Kuala Lumpur, Malaysia; 4 Clinical Investigation Centre, University Malaya Medical Centre, Kuala Lumpur, Malaysia; 5 Department of Pathology, Faculty of Medicine, University of Malaya, Kuala Lumpur, Malaysia; 6 Saw Swee Hock School of Public Health, National University of Singapore, Singapore, Singapore; Wayne State University, United States of America

## Abstract

Angiotensin II type 1 receptor (*AGTR1*) has been reported to play a fibrogenic role in non-alcoholic fatty liver disease (NAFLD). In this study, five variants of the *AGTR1* gene (rs3772622, rs3772627, rs3772630, rs3772633, and rs2276736) were examined for their association with susceptibility to NAFLD. Subjects made up of 144 biopsy-proven NAFLD patients and 198 controls were genotyped using TaqMan assays. The liver biopsy specimens were histologically graded and scored according to the method of Brunt. Single locus analysis in pooled subjects revealed no association between each of the five variants with susceptibility to NAFLD. In the Indian ethnic group, the rs2276736, rs3772630 and rs3772627 appear to be protective against NAFLD (*p* = 0.010, *p* = 0.016 and *p* = 0.026, respectively). Haplotype ACGCA is shown to be protective against NAFLD for the Indian ethnic subgroup (*p* = 0.03). Gene-gene interaction between the *AGTR1* gene and the patatin-like phospholipase domain-containing 3 (*PNPLA3*) gene, which we previously reported as associated with NAFLD in this sample, showed a strong interaction between *AGTR1* (rs3772627), *AGTRI* (rs3772630) and *PNPLA3* (rs738409) polymorphisms on NAFLD susceptibility (*p* = 0.007). Further analysis of the NAFLD patients revealed that the G allele of the *AGTR1* rs3772622 is associated with increased fibrosis score (*p* = 0.003). This is the first study that replicates an association between *AGTR1* polymorphism and NAFLD, with further details in histological features of NAFLD. There is lack of evidence to suggest an association between any of the five variants of the *AGTR1* gene and NAFLD in the Malays and Chinese. In the Indians, the rs2276736, rs3772630 and rs3772627 appear to protect against NAFLD. We report novel findings of an association between the G allele of the rs3772622 with occurrence of fibrosis and of the gene-gene interaction between *AGTR1*gene and the much-studied *PNPLA3* gene.

## Introduction

The studies on non-alcoholic fatty liver disease (NAFLD) have rapidly increased since the term non-alcoholic steatohepatitis was first introduced by Ludwig et al [1]. NAFLD, defined as fatty accumulation of the liver without significant alcohol intake, ranges from simple steatosis to non-alcoholic steatohepatitis (NASH) and cirrhosis [2]. The histological features of NAFLD include steatosis, lobular inflammation, hepatocellular ballooning and fibrosis [3]. NAFLD is the hepatic manifestation of metabolic syndrome [4]–[8]. Although environmental factors, lifestyle and dietary intake underlie susceptibility to NAFLD, genetic factors play a significant upstream role [9]–[11].

The renin-angiotensin system acts on the hepatic stellate cells (HSC) to promote hepatic fibrosis [12]–[13]. The *AGTR1* gene has been implicated with susceptibility to NAFLD [13]–[14]. To date, there is only one study of *AGTR1* polymorphism and its association with the occurrence of NAFLD. The single nucleotide polymorphisms (SNPs) are located in the intron of *AGTR1* on chromosome 3. In the study, five variants of *AGTR1*(rs3772622, rs3772627, rs3772630, rs3772633, and rs2276736) were revealed to be strongly associated with risk of NAFLD [15]. Furthermore, the deletion of angiotensin II type 1 receptor in animals was associated withdecreased hepatic steatosis. Thus, *AGTR1* may be an important regulator of steatosis in the liver [16].

The data on *AGTR1* gene polymorphisms and its association with NAFLD is currently limited to one study, therefore, we investigated the association between five previously reported SNPs of the *AGTR1* gene with NAFLD susceptibility, in three major ethnic subgroups of the Malaysian population, namely the Malays, Chinese and Indians. These three ethnic groups, which are presumably of different genetic pools, provide a good setting to study possible ethnic differences in susceptibility to NAFLD. We further evaluated the association between the SNPs mentioned and the histological features of NAFLD, namely steatosis, lobular inflammation, hepatocellular ballooning and fibrosis. We previously reported in this same subjects, the association between *PNPLA3* with NAFLD and disease severity [17]. In this study, we report new findings for the interaction between the *AGTR1* gene and the *PNPLA3* gene on susceptibility to NAFLD.

## Methods

### Ethics Statement

Subjects provided written informed consent after a full explanation of the research outline. The study protocol was reviewed and approved by the Medical Ethics Committee of University Malaya Medical Centre (UMMC).

### Subjects

A case control study conducted at the UMMC recruited a total of 144 cases and 198 controls. All cases were biopsy-proven NAFLD patients based on increased echogenicity (compared to renal cortex) on ultrasound with or without abnormal alanine transferase (ALT). Ethnicity of the subjects were confirmed by affirmations of absence of mixed marriages for at least three generations.Exclusion criteria included alcohol consumption > 10g/day [18], hepatitis B or C infection, autoimmune hepatitis, those who had been on drugs that are known to cause steatosis, and Wilson's disease. All the biopsy specimens were on an average 1.5 cm long and contained six portal tracts. The biopsy specimens were graded according to the method of Brunt [19]–[20]. The controls were genetically unrelated healthy subjects presenting with body mass index (BMI) <25 kg/m^2^, fasting plasma glucose <110 mg/dL, normal lipid profile and normal liver enzymes. NAFLD was excluded in the controls by ultrasonography according to the absence of the following criteria; diffuse increase in bright echoes in the liver parenchyma (exceeding that of renal cortex and spleen) with impaired visualization of the peripheral portal and hepatic vein borders, and/or loss of definition of the diaphragm and poor delineation of the portal and hepatic radicles.

### Clinical and laboratory assessments

Venous blood samples were taken for the determination of haemoglobin A1c (HbA1c), total cholesterol, triglycerides, high-density lipoprotein cholesterol (HDL), low-density lipoprotein cholesterol (LDL), alanine transferase (ALT), aspartate aminotransferase (AST), and gamma-glutamyl transpeptidase (GGT) level in all subjects. The biochemical tests were analysed based on standard clinical laboratory methods. Other relevant parameters such as body mass index (BMI), waist circumference and blood pressure weredetermined according to standard protocol.

### Genotyping

Blood samples were collected in EDTA tubesandgenomic DNA was prepared using the QiAamp DNA Mini Kit (Qiagen. Hilden, German) according to user protocol. The *AGTR1* SNPs were genotyped using a predesigned TaqMan SNP genotyping assay (Applied Biosystems, Foster City, CA, USA) as previously described [17].

### Statistical analyses

All values are presented as mean±standard deviation for continuous data and as percentages for categorical data. For normally distributed variables (age, BMI, waist circumference, ALT, HDL cholesterol, LDL cholesterol, total cholesterol, triglycerides, and systolic blood pressure), independentt-test was performed to determine the associations between the different grades of NAFLD. For variables that are not normally distributed (HbA1c, AST, GGT, diastolic blood pressure, steatosis grade, lobular inflammation, ballooning, and fibrosis), Mann-Whitney U test was performed. Hardy-Weinburg equilibirum (HWE) was checked for all the groups using a goodness-of fit χ2 test with one degree of freedom. A *p* value of less than 0.05 indicated a lack of agreement with HWE.

Association of allele was performed using logistic regression. Likelihood tests indicated a significant effect of ethnicity but no significant effect of age and gender. In order to avoid false discoveries due to population stratification, the association analysis was performed for each marker (SNP/haplotype) separately for each ethnic group. To come up with an overall estimate, the estimates from the different ethnic groups were combined using inverse variance weighting (IVW) technique, usually used for combining estimates in meta-analysis. The calibration and fit of the model were assessed using Hosmer Lemeshow goodness of fit and receiver operating characteristic (ROC) curves. Kruskal-Wallis test was performed for comparison of medians between three groups.

To investigate the influence of gene-gene interaction on NAFLD, Generalized Multifactor Dimensionality Reduction (GMDR) method was employed [21]. All possible interactions were tested using 10-fold cross validation with exhaustive search, which considers all possible variable combinations. GMDR provides a cross-validation consistency score which is a measure of the degree of consistency with which the selected interaction is identified as the best model among all possibilities considered. The testing balanced accuracy generated is a measure of the degree to which the interaction accurately predicts case-control status. Testing accuracy is a measure of the strength of gene-gene interaction with a power of 80% at an accuracy of 0.58–0.60, given a sample of <500 [22]. In addition, logistic regression model was performed to confirm the results of gene interaction analyses. Ethnicity was used as covariate in the gene-gene interaction analysis.

Univariate analysis of association of genotypes and histological ordinal variables was assessed using the Jonckheere-Terpstra test. Ordinal regression is perfomed for multivariate analysis of histological ordinal variables. To correct for testing for multiple histological parameters, the false discovery rate (FDR) was calculated using Benjamini-Hochberg procedure[23]. Analyses were performed using SPSS 16.0 (Chicago IL) with a two sided *p*< 0.05 considered to be statistically significant. Linkage disequilibrium and haplotype analyses for the five SNPs were performed using Haploview 4.2 program. The odds ratio for the haploypes was calculated using R software version 2.11.1.

We estimated that a sample size of 117 cases and 161 controls would provide 80% power at α of 0.05 with the following assumptions: the allele frequency range from 0.31-0.41, the baseline risk for the Malaysian population was 0.17, and the minimum detectable odds ratio was 2.0.

## Results

Table 1 shows the demographic and clinical data of the NAFLD case (n = 144) and the control (n = 198) groups. There were 59 (41%) Malays, 54 (38%) Chinese and 31 (21%) Indians in the NAFLD group. Out of the 198 controls, 80 (41%) were Malays, 54 (27%) Chinese and 64 (32%) Indians. The differences in the parameters of BMI, HbA1c, LDL cholesterol, total cholesterol, triglycerides, AST, ALT and GGT between the two groups reflect the nature of the selection of the cases and controls.

**Table 1:** Demographic and clinical data of the subjects.

Characteristics	*n* (%) or Mean±SD		
	Control (*n* = 198)	NAFLD (*n* = 144)	*p* value
Gender			0.084
Males	85 (43)	77 (53)	
Females	113 (57)	67 (47)	
Ethnicity			
Malays	80 (41)	59 (41)	
Chinese	54 (27)	54 (38)	
Indians	64 (32)	31 (21)	
Age (years)	53.1±11.5	51.2±12.0	0.136
BMI (kg/m^2^)	22.7±2.6	28.7±4.4	<0.0001
HbA1c (%)	5.7±0.8	6.6±1.7	<0.0001
HDL cholesterol (mg/dl)	49.5±12.9	48.5±12.7	0.31
LDL cholesterol (mg/dl)	89.5±22.4	117.1±40.0	<0.0001
Total cholesterol (mg/dl)	176.4±26.9	196.7±44.0	<0.0001
Triglycerides (mg/dl)	118.4±32.3	155.0±62.7	<0.0001
AST (IU/L)	21.8±9.5	42.9±25.4	<0.0001
ALT (IU/L)	36.0±16.6	83.0±48.5	<0.0001
GGT (IU/L)	44.0±25.4	111.6±115.5	<0.0001

Data are expressed as mean±SD for continuous data and as percentage for categorical data.

*ALT* alanine transferase, *AST* aspartate aminotransferase, *BMI* body mass index, *GGT* gamma glutamyl transpeptidase, *HbA1c* haemoglobin A1c, *HDL* high-density lipoprotein, *LDL* low-density lipoprotein, *NAFLD* non-alcoholic fatty liver disease.

*p* values obtained using Mann-Whitney U test.

Table 2 further describes the demographic and clinical data of the NAFLD subjects grouped into simple steatosis (n = 33) and NASH (n = 111). The BMI, HbA1c, waist circumference, triglycerides, systolic, and diastolic blood pressure were significantly higher in the NASH group (*p*<0.05) as compared to the simple steatosis group.

**Table 2:** Demographic and clinical data of the NAFLD patients.

Characteristics	*n* (%) or Mean±SD		
	Simple steatosis(n = 33)	NASH(n = 111)	*p* value
Gender, n (%)			0.461
Males	20 (61)	57 (51)	
Females	13 (39)	54 (49)	
Age (years)	50.7±11.8	51.2±12.1	0.82
BMI (kg/m^2^)	26.7±3.9	29.2±4.4	0.003
HbA1c (%)*	6.1±1.3	6.7±1.7	0.021
Waist circumference (cm)	89.2±11.2	95.2±10.4	0.005
HDL cholesterol (mg/dl)	50.2±15.1	48.0±11.9	0.391
LDL cholesterol (mg/dl)	114.7±42.8	117.8±39.3	0.698
Total cholesterol (mg/dl)	191.6±44.9	198.2±43.8	0.448
Triglycerides (mg/dl)	124.6±42.0	164.1±65.1	0.001
AST (IU/L)*	37.6±21.3	44.5±26.3	0.139
ALT (IU/L)	71.9±50.0	86.4±47.8	0.134
GGT (IU/L)*	99.4±106.6	115.3±118.2	0.132
Systolic blood pressure (mmHg)	125.2±13.0	134.2±14.1	0.001
Diastolic blood pressure (mmHg)*	78.2±9.1	83.8±9.7	0.003

*ALT* alanine transferase, *AST* aspartate aminotransferase, *BMI* body mass index, *GGT* gamma glutamyl transpeptidase, *HbA1c* haemoglobin A1c, *HDL* high-density lipoprotein, *LDL* low-density lipoprotein, *NAFLD* non-alcoholic fatty liver disease, *NASH* non-alcoholic steatohepatitis

* *P* values obtained using Mann-Whitney U test, all other comparisons used independent *t* test

### Genotypes and allele frequencies of *AGTR1* polymorphisms

The genotypes of each SNP were in Hardy-Weinberg equilibrium for both NAFLD cases and controls, for pooled subjects as well as after stratification by ethnicity. The association tests between NAFLD and control subjects are shown in Table 3. None of the SNPs were associated with susceptibility to NAFLD. However, after ethnic stratification, in the Indian ethnic subgroup, the rs2276736, rs3772630 and rs3772627 were found to be protective against NAFLD (OR 0.40, 95% CI 0.20–0.81, *p* = 0.010; OR 0.43, 95% CI 0.22–0.86, *p* = 0.016; and OR 0.46, 95% CI 0.23–0.91, *p* = 0.026, respectively). We showed the sample power for the significant findings to be > 81%.

**Table 3:** Association tests of *AGTR1* SNPs and NAFLD.

NAFLD spectrum	All ethnicities*			Malay			Chinese			Indian		
	MAF	OR (CI)	*p*	MAF	OR (CI)	*p*	MAF	OR (CI)	*p*	MAF	OR (CI)	*p*
rs2276736(T>C)**												
*Control as reference*	0.42	1.00		0.35	1.00		0.43	1.00		0.49	1.00	
NAFLD	0.38	0.82 (0.48–1.37)	0.445	0.36	1.03 (0.61–1.15)	0.917	0.44	1.08 (0.62–1.88)	0.778	0.27	0.40 (0.20–0.81)	0.010
Simple steatosis	0.41	0.99 (0.55–1.79)	0.968	0.33	0.93 (0.37–2.31)	0.873	0.5	1.35 (0.62–2.93)	0.450	0.25	0.35 (0.07–1.81)	0.209
NASH	0.36	0.78 (0.46–1.34)	0.366	0.36	1.05 (0.61–1.81)	0.848	0.42	0.97 (0.53–1.78)	0.925	0.3	0.42 (0.21–0.86)	0.017
rs3772622 (A>G)												
*Control as reference*	0.38	1.00		0.38	1.00		0.41	1.00		0.34	1.00	
NAFLD	0.42	1.17 (0.87–1.59)	0.301	0.45	1.30 (0.81–2.07)	0.275	0.4	0.97 (0.58–1.61)	0.896	0.4	1.32 (0.69–2.53)	0.409
Simple steatosis	0.38	0.97 (0.57–1.65)	0.912	0.33	0.83 (0.35–1.97)	0.666	0.38	0.91 (0.43–1.94)	0.804	0.5	1.96 (0.45–8.55)	0.371
NASH	0.43	1.22 (0.88–1.69)	0.226	0.48	1.46 (0.88–2.42)	0.142	0.41	0.99 (0.58–1.71)	0.981	0.39	1.24 (0.62–2.46)	0.546
rs3772633 (A>G)												
*Control as reference*	0.23	1.00		0.23	1.00		0.3	1.00		0.19	1.00	
NAFLD	0.28	1.21 (0.86–1.70)	0.282	0.28	1.28 (0.75–2.18)	0.370	0.34	1.23 (0.70–2.16)	0.475	0.19	1.04 (0.49–2.21)	0.922
Simple steatosis	0.24	0.99 (0.53–1.84)	0.965	0.17	0.66 (0.21–2.08)	0.473	0.35	1.27 (0.58–2.80)	0.550	0.12	0.61 (0.07–5.34)	0.656
NASH	0.3	1.31 (0.91–1.88)	0.150	0.31	1.45 (0.83–2.55)	0.190	0.34	1.20 (0.65–2.23)	0.564	0.22	1.23 (0.57–2.66)	0.597

* Results based on combining results across ethnicities

** Only results of rs2276736 presented due to very high LD of rs2276736, rs3772630, rs3772627. The results for rs3772630 and rs3772627 are similar to rs2276736 (Table S1)

*CI* confident interval, *MAF* minor allele frequency, *NAFLD* non-alcoholic fatty liver disease, *NASH* non-alcoholic steatohepatitis, *OR* odds ratio

We then divided the NAFLD group into simple steatosis and NASH but did not find any association between the five SNPs with susceptibility to NASH in the pooled subjects (Table 3). Results by ethnicity group show that in the Indian ethnic subgroup, the three SNPs (rs2276736, rs3772630 and rs3772627) were also protective against NASH (OR 0.42, 95% CI 0.21–0.86, *p* = 0.017; OR 0.46, 95% CI 0.22–0.92, *p* = 0.029; and OR 0.49, 95% CI 0.24–0.98, *p* = 0.045, respectively).

### Gene-gene interaction analysis

The overall results, pooling all subjects, indicate a lack of evidence for an association between*AGTR1* gene and NAFLD, which is in contrast with the positive findings by Yoneda et. al [15]. Our previous study showed a strong association of *PNPLA3* gene with the occurence of NAFLD [17], and we were therefore curious to find out whether there is any gene-gene interaction between *PNPLA3* and *AGTR1*. The results with the two-locus and three-locus models are shown in Table 4. The two-locus model has the highest testing accuracy while the three-locus model has the best cross-validation consistency. Investigation of the gene-gene interaction on the occurence of NAFLD, identified a significant effect of *PNPLA3* and *AGTR1* interaction (empirical *p* = 0.007). The best GMDR model suggested for the interaction was the three-locus model. The significant interaction was confirmed by logistic regression (permuted *p* = 0.017).

**Table 4:** Best fitted gene-gene interaction model.

Locus number	Model	Cross-validation consistency	Testing accuracy (%)	*p*value*
2	*AGTR1* (rs2276736), *PNPLA3* (rs738409)	8/10	63.68	0.038
3	*AGTR1* (rs3772627), *AGTRI* (rs3772630), *PNPLA3* (rs738409)	9/10	62.88	0.007

* *P* values based on 1000 permutations. Analysis of GMDR with adjustment of ethnicity

Genotype association in additive model revealed that the risk genotypes (*AGTR1* rs3772627TT, *AGTR1* rs3772630AA and *PNPLA3* rs738409GG) versus one risk factor (*AGTR1* rs3772627TC, *AGTR1* rs3772630AG and *PNPLA3* rs738409CG) versus the protective genotypes (*AGTR1* rs3772627CC, *AGTR1* rs3772630GG and *PNPLA3* rs738409CC), conferred enhanced risk for NAFLD (OR 2.23, 95% CI 1.16–4.31, *p* = 0.017). Since the protective effect was seen in the Indian ethnic subgroup, we additionally tested the association specifically in this group. The risk for NAFLD in this group was dramatically enhanced (OR 12.41, 95% CI 1.61–55.42), *p* = 0.016) following genotypes combinations. We showed that the gene-gene interaction with an effect size of 4.5 would provide a power of > 79%. We further evaluated the association of *AGTR1* SNPs with NAFLD following stratification by *PNPLA3* genotypes. The results indicated the absence of significant association (data not shown).

### *AGTR1* polymorphisms and liver histology

**Figure 1:**
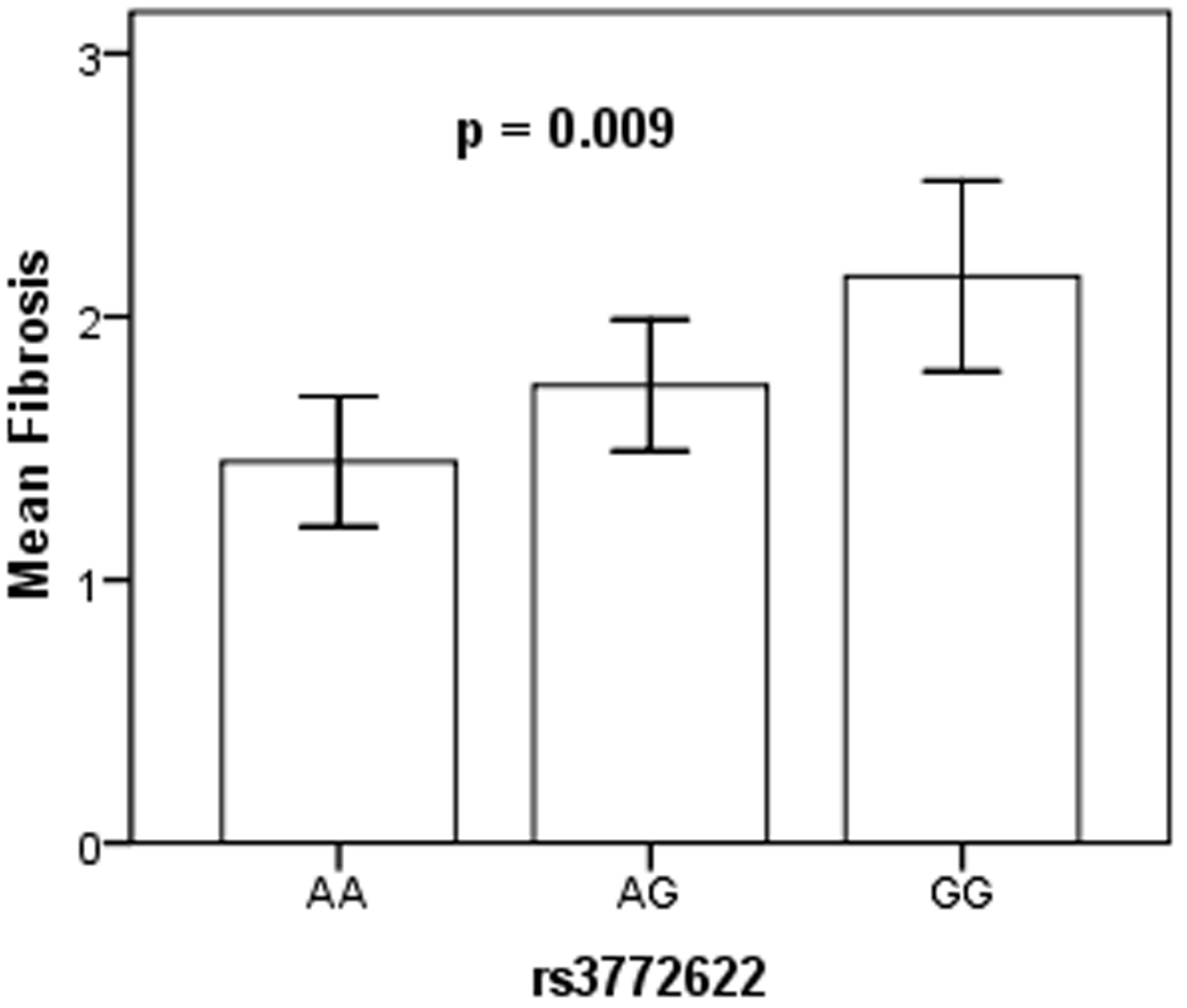
Mean fibrosis score among the genotypes of rs3772622. *p* value calculated from comparison between three groups using Kruskal-Wallis test.

Of the five SNPs, rs3772622 was the only SNP to show association with liver histology in the NAFLD patients (Table 5). The GG genotype of this SNP is significantly associated with increased in fibrosis (*p* = 0.009) (Fig. 1). It has to be noted that the rs3772622 is not among the SNPs that have been found to be protective. To investigate whether the G allele of the rs3772622 is associated with risk of fibrosis, we performed univariate and multivariate tests. The G allele of this SNP was shown to be associated with presence of fibrosis (*p* = 0.003). Subjects with the G allele has a propensity of 2.18 times to develop fibrosis score ≥ 2. The association remains significant (*p* = 0.003) after adjustment of other histological attributes (Table 5). We also investigated the remaining SNPs and their association with histological features of NAFLD, however none of them showed a significant association (data not shown).

**Table 5:** Association of G allele of rs3772622 with histological features in NAFLD patients.

Histology	Univariate*p*value^a^	multivariate *p*value^b^(FDR *q*value^g^)	OR (95% CI)
Steatosis>33% (n = 85) vs. <33% (n = 59)	0. 589	0.546 (0.728)	0.98 (0.61–1.56)^c^
Lobular inflammation≥2 foci (n = 51) vs. <2 foci (n = 93)	0. 953	0.251 (0.502)	0.89 (0.55–1.45)^d^
Hepatocellular ballooning≥1 (n = 131) vs. <1 (n = 13)	0. 395	0.744 (0.744)	2.08 (0.84–5.14)^e^
Fibrosis≥2 (n = 76) vs. <2 (n = 68)	0. 003	0.003 (0.012)	2.18 (1.32–3.60)^f^

*OR* odds ratio, *CI* confident interval

a Jonckheere-Terpstra test

b Ordinal regression

c, d, e, f Multivariate logistic regression

g False discovery rate, q<0.05 is significant

### Linkage disequilibrium and haplotype analysis of *AGTR1* SNPs

Linkage disequilibrium (LD) analysis revealed a strong LD between the five SNPs in overall subjects and after ethnic stratification except between rs3772633 and rs3772622. Variants rs2276736 and rs3772630 are in complete LD in the Chinese and Malays, however variants rs2276736 and rs3772627 are in complete LD in the Chinese only (Table 6).

**Table 6:** Linkage disequilibrium coefficient among five SNPs of *AGTR1* gene.

SNP ID	D′		Chinese	
	rs2276736	rs3772630	rs3772627	rs3772622
rs3772633	0.82	0.81	0.82	0.68
rs2276736		1.00	1.00	0.84
rs3772630			0.96	0.78
rs3772627				0.82
SNP ID	D′		Indian	
	rs2276736	rs3772630	rs3772627	rs3772622
rs3772633	0.81	0.80	0.87	0.52
rs2276736		0.96	0.98	0.91
rs3772630			0.98	0.86
rs3772627				0.85
SNP ID	D′		Malay	
	rs2276736	rs3772630	rs3772627	rs3772622
rs3772633	0.90	0.90	0.90	0.89
rs2276736		1.00	0.98	0.86
rs3772630			0.95	0.87
rs3772627				0.81
SNP ID	D′		All Ethnicities	
	rs2276736	rs3772630	rs3772627	rs3772622
rs3772633	0.85	0.85	0.86	0.73
rs2276736		0.99	0.98	0.86
rs3772630			0.95	0.83
rs3772627				0.85

*D*′ linkage disequilibrium coefficient, *SNP* single nucleotide polymorphism

Four haplotypes with frequencies of above 5% are presented in Table 7. In pooled subjects, haplotypes ATATG, GCGCA and ATATA are more frequent than the others (35.1%, 21.8% and 20.9%, respectively). Similar results are observed in the Malays (37.8%, 22.7% and 23.2%, respectively). Meanwhile in the Chinese, the most frequent haplotypes are ATATG (34.8%) and GCGCA (25.9%). The Indians on the other hand has high frequency of haplotypes ATATG (31.4%), ATATA (22.3%) and ACGCA (24.7%). Each of the following haplotypes, ATATG, GCGCA, ATATA and ACGCA is significantly different among the ethnic groups (*p*< 0.0001, for each haplotype respectively). Haplotype analysis revealed that haplotype ACGCA was protective against NAFLD in the Indians (OR 0.42, 95% CI 0.15-0.95, *p* = 0.03). Since rs2276736, rs3772630 and rs3772627 are presented in similar structure and some were in complete LD, we further analysed the haplotype among the three SNPs. We found that the Indians presenting with the CGC haplotype were protected (OR 0.41, 95% CI 0.21–0.84, *p* = 0.015) against NAFLD (data not shown).

**Table 7:** Haplotype frequencies of *AGTR1* SNPs.

Haplotype	Ethnicity															
	Chinese (n = 108)				Indian (n = 95)				Malay (n = 139)				Total (n = 342)			
	Case (%)	Ctrl (%)	p	OR^a^(95% CI)	Case (%)	Ctrl (%)	p	OR^a^ (95% CI)	Case (%)	Ctrl (%)	p	OR^a^ (95% CI)	Case (%)	Ctrl (%)	p^c^	OR^a^ (95% CI)
ATATG	33	37	0.60	Referent	33	31	0.75	Referent	43	34	0.15	Referent	37	34	0.37	Referent
GCGCA	26	26	0.99	1.07 (0.54–2.10)	12	18	0.28	0.59 (0.20–1.68)	24	22	0.59	0.74 (0.38–1.44)	22	22	0.40	0.83 (0.54–1.28)
ATATA	16	18	0.67	1.02 (0.45–2.32)	31	18	0.05	1.62 (0.59–3.28)	18	27	0.09	0.54 (0.29–1.03)	20	22	0.62	0.86 (0.48–1.54)
ACGCA	15	14	0.84	1.26 (0.53–2.96)	15	29	0.03	0.42 (0.15–0.95)	8	12	0.34	0.53 (0.22–1.28)	13	18	0.23	0.66 (0.34–1.29)
Others^b^	10	5	—	—	9	4	—	—	7	5	—	—	8	4	—	—
Total	100	100	—	—	100	100	—	—	100	100	—	—	100	100	—	—

*CI* confidence interval, *Ctrl* control, *OR* odds ratio, ^a^OR estimated odds ratio by R, ^b^Haplotypes with total frequencies below 5% in all subjects, ^c^ *P* values based on combining results across ethnicities

## Discussion

Angiotensin II appears to have an important role in the progression to non-alcoholic steatohepatitis (NASH) [13]–[14]. Angiotensin II acts on angiotensin II type 1 receptor to activate hepatic stellate cells (HSCs). The activated HSCs will express the transforming growth factor-β 1 (TGF-β 1), a profibrogenic cytokine, as a counter response to liver injury [12]–[13]. The preventive role of *AGTR1* blocker in the progression to NASH was succesfully observed in the animaland human studies [24]–[27]. The deletion of angiotensin II type 1 receptor in animal studies has been shown to reduce hepatic steatosis suggesting *AGTR1* is an important regulator of hepatic steatosis [16].

In the present study, our data indicated that the five previously reported SNPs of the angiotensin II type I receptor (*AGTR1*) gene (rs3772622, rs3772627, rs3772630, rs3772633, and rs2276736) were not associated with susceptibility to non-alcoholic fatty liver disease (NAFLD) and non-alcoholic steatohepatitis (NASH). This is in contrast with the Japanese study which showed that these *AGTR1* polymorphisms were significantly associated with the occurence of NAFLD and NASH [15].

Among the SNPs studied, rs2276736, rs3772630 and rs3772627 were found to be protective against NAFLD and NASH in the Indian ethnic subgroup. Interestingly, these three SNPs share the same linkage disequilibrium structure. We revealed that haplotype ACGCA is protective against NAFLD in the Indians. The study by Yoneda et al reported that the protective haplotype in the Japanese was ATATG [15]. The discrepancy in the findings may be explained by ethnic differences between the populations studied. The three major ethnic subgroups in Malaysia, namely the Malay, Chinese and Indian are of the south Asian origin with the Chinese are of the Hans descendant of Southern China [28], Indians from Southern India [29], whereas the Malays are the indigenous ethnic group. There is a supportive evidence that the Japanese are from the north Asian (Northern China and Korea) origin but not the south Asian (Southern China and Southeast Asia) based on the classical marker polymorphisms, Y chormosome and mitochondrial DNA [30]–[31]. The difference in the association studies between the two population probably reflects the difference in the genetic pools of the Malaysian and the Japanese.

The results from our study indicated that there was no difference in levels of liver enzymes between patients with simple steatosis and those with NASH. This is in agreement with the results from Sorrentino et al [32] and Mofrad et al [33], but is in contrast with the findings of several other studies which showed differences in levels between the two groups [34]–[37]. The differences in these findings is probably due to the differences in the recruitment of subjects for the various studies, in which inclusion criteria ranges from morbid obese NAFLD to adults with NAFLD. Furthermore, we observed a significantly higher triglycerides levels in patients with NASH compared to those with simple steatosis. Triglyceride storage in hepatocytesmarks the intensity of exposure to potentially toxic fatty acids. As NAFLD progresses, excess free fatty acids are compensated with increase in triglycerides production [38].

Analysis of interaction between *PNPLA3* and *AGTR1* gene, revealed a strong interaction between *AGTR1* (rs3772627), *AGTRI* (rs3772630) and *PNPLA3* (rs738409) SNPs on NAFLD susceptibility. Compared to the Malays and Chinese, the Indians presented with the greatest susceptibilty to NAFLD in the *PNPLA3* study [17]. Thus the *PNPLA3*can possibly mask the effect of *AGTR1* on NAFLD among the Indians. Although both genes encode different products (*PNPLA3* encodes adiponutrin while *AGTR1* encodes angiotensin II type 1 receptor), the strong interaction observed suggested a possible interaction as both genes are involved in lipid accumulation. The action of angiotensin II on angiotensin II type 1 receptor (*AGTR1*) promotes lipid accumulation in the liver whilelack of *AGTR1* reduces hepatic triglycerides [16]. Adiponutrin (*PNPLA3*) on the other hand is highly upregulated in response to feeding and downregulated in fasting state, displaying its role in lipid storage in the liver [39]. Of note, *AGTR1* and *PNPLA3* are correlatively upregulated in a state of excess lipid.

We further showed that the SNP rs3772622 was associated with occurence of fibrosis but not with the other histological parameters of NAFLD. The G allele of this SNP is associated with increased fibrosis score. The Japanese also found that SNP rs3772622 was associated with the presence of fibrosis [15], but in contrast to our findings, the G allele was found to be associated with decreased fibrosis score. The difference is probably explained by a higher minor allele frequency in our population than that of the Japanese. A greater systolic blood pressure mean value (136.00 mmHg) observed in the GG genotype compared to the AA genotype (128.65 mmHg) of the SNP rs3772633 could also contribute to the discrepancy. Data of the previous report showed that hypertensive patients have significantly higher fibrosis score than that of non-hypertensive [40]. The strong linkage disequilibrium observed between the five SNPs in the overall subjects and even after ethnic stratification indicates a potential tagSNP selection based on the pairwise LD. This approach will be useful in future in order to minimize the genotyping cost yet presenting the information that could reflect the role of the other relevant SNPs within the gene.

This present study has several limitations and strengths. Studies on NAFLD spectrum have been limited since this requires valid ascertainment and scoring of the different NAFLD stages, for which liver biopsy is the gold standard in providing such accurate diagnosis. Such biopsies are limited to situations when there is a definite clinical indication for the biopsy, and this reflects the relatively small sample size in our study. Unlike the diagnosis of NASH, diagnosis of fatty liver can be made based on clinical, radiological and laboratory findings, which therefore makes it slightly easier to carry out studies on NAFLD. Ethics consideration does not allow for biopsy in control subjects, thus no biopsies were performed in controls. In order to reduce likelihood of misclassifying the controls, a strict criteria are required. These criteria includes normal BMI, fasting plasma glucose, lipid profile and liver enzymes. The difference in the distribution of these parameters between the two extremes of the NAFLD disease spectrum, provides confidence in the selection process of NAFLD cases and controls. Mild steatosis among the controls cannot however be ruled out and this may result in a bias towards the null. The resulting association between *AGTR1* gene and NAFLD was smaller than hypothesized (OR less than 2.0) and would have resulted in a loss of power for the relevant analysis. The small sample size after stratification into simple steatosis and NASH is another limitation for this study. A major strength of this study was the ability to compare the association between *AGTR1* polymorphisms and NAFLD among three major ethnic groups in Asia. This study suggest that the rs2276736, rs3772630 and rs3772627 are protective against NAFLD among the Indians. This study is also the first to report the interaction of gene between the *AGTR1* and the commonly reported *PNPLA3* gene on NAFLD susceptibility.

In conclusion, the present study suggests that the *AGTR1* variants rs2276736, rs3772630 and rs3772627 are protective against NAFLD among the Indians. The G allele of rs3772622 is significantly associated with presence of fibrosis. Among the Malays and Chinese, we failed to find any significant association between the five SNPs (rs3772622, rs3772627, rs3772630, rs3772633, and rs2276736) of *AGTR1* gene and susceptibility to NAFLD. We report novel finding for the gene-gene interaction between *AGTR1* and the much-studied *PNPLA3* genes. A larger sample size and more ethnically diverse samples are needed to replicate these findings.

## Supporting Information

Table S1 **Association tests of** ***AGTR1*** **SNPs and NAFLD.**
(DOCX)
