# Supplementary material for: Susceptibility and Gene Interaction Study of the Angiotensin II Type 1 Receptor (AGTR1) Gene Polymorphisms with Non-Alcoholic Fatty Liver Disease in a Multi-Ethnic Population
Source: PLoS One. 2013 Mar 6;8(3):e58538. doi: 10.1371/journal.pone.0058538 (PMC3590220; doi:10.1371/journal.pone.0058538)
Supplement: Table S1 — Association tests of AGTR1 SNPs and NAFLD. (DOCX) [file pone.0058538.s001.docx]

**Table S1** Association tests of *AGTR1* SNPs and NAFLD

| NAFLD spectrum | All ethnicities* | | |  | Malay | | |  | Chinese | | |  | Indian | | |
| --- | --- | --- | --- | --- | --- | --- | --- | --- | --- | --- | --- | --- | --- | --- | --- |
|  | MAF | OR (CI) | *p* |  | MAF | OR (CI) | *p* |  | MAF | OR (CI) | *p* |  | MAF | OR (CI) | *p* |
| rs3772630 (A > G) |  |  |  |  |  |  |  |  |  |  |  |  |  |  |  |
| *Control as reference* | 0.42 | 1.00 |  |  | 0.35 | 1.00 |  |  | 0.44 | 1.00 |  |  | 0.49 | 1.00 |  |
| NAFLD | 0.39 | 1.03 (0.76-1.38) | 0.859 |  | 0.37 | 1.10 (0.67-1.81) | 0.695 |  | 0.45 | 1.08 (0.62-1.88) | 0.779 |  | 0.31 | 0.43 (0.22-0.86) | 0.016 |
| Simple steatosis | 0.42 | 1.01 (0.73-1.39) | 0.951 |  | 0.38 | 1.11 (0.46-2.67) | 0.813 |  | 0.50 | 1.31 (0.60-2.87) | 0.504 |  | 0.25 | 0.35 (0.07-1.81) | 0.209 |
| NASH | 0.38 | 1.09 (0.67-1.78) | 0.728 |  | 0.37 | 1.10 (0.65-1.88) | 0.719 |  | 0.43 | 0.99 (0.54-1.80) | 0.971 |  | 0.31 | 0.46 (0.22-0.92) | 0.029 |
| rs3772627 (T > C) |  |  |  |  |  |  |  |  |  |  |  |  |  |  |  |
| *Control as reference* | 0.42 | 1.00 |  |  | 0.38 | 1.00 |  |  | 0.44 | 1.00 |  |  | 0.48 | 1.00 |  |
| NAFLD | 0.38 | 0.62 (0.33-1.15) | 0.128 |  | 0.36 | 0.92 (0.56-1.52) | 0.742 |  | 0.45 | 1.08 (0.63-1.85) | 0.783 |  | 0.31 | 0.46 (0.23-0.91) | 0.026 |
| Simple steatosis | 0.41 | 0.63 (0.38-1.04) | 0.073 |  | 0.33 | 0.84 (0.34-2.06) | 0.697 |  | 0.50 | 1.29 (0.60-2.76) | 0.516 |  | 0.25 | 0.37 (0.07-1.92) | 0.236 |
| NASH | 0.37 | 0.71 (0.28-1.82) | 0.473 |  | 0.36 | 0.94 (0.55-1.61) | 0.831 |  | 0.43 | 0.99 (0.55-1.78) | 0.971 |  | 0.31 | 0.49 (0.24-0.98) | 0.045 |

* Results based on combining results across ethnicities

*CI* confident interval, *MAF* minor allele frequency, *NAFLD* non-alcoholic fatty liver disease,*NASH* non-alcoholic steatohepatitis, *OR* odds ratio
